# Transcriptional Regulation of the *Acer truncatum* B. Response to Drought and the Contribution of *AtruNAC36* to Drought Tolerance

**DOI:** 10.3390/antiox12071339

**Published:** 2023-06-24

**Authors:** Jianbo Li, Wei Guo, Jinna Zhao, Huijing Meng, Yanfei Yang, Guangshun Zheng, Weijie Yuan

**Affiliations:** 1 Experimental Centre of Forestry in North China, Chinese Academy of Forestry, Beijing 102300, China; lijb2017@caf.ac.cn (J.L.); jinnazz@163.com (J.Z.); 15938277261@163.com (H.M.); 13759225665@163.com (Y.Y.); zhengguangshun@caf.ac.cn (G.Z.); 2 National State Key Laboratory of Tree Genetics and Breeding, Chinese Academy of Forestry, Beijing 100091, China; 3 Taishan Academy of Forestry Sciences, Tai’an 271000, China; guowei@ta.shandong.cn

**Keywords:** *Acer truncatum*, drought, *AtruNAC36*, antioxidant enzyme

## Abstract

Drought stress is one of the major environmental factors severely restricting plant development and productivity. *Acer truncatum* B, which is an economically important tree species, is highly tolerant to drought conditions, but the underlying molecular regulatory mechanisms remain relatively unknown. In this study, *A. truncatum* seedlings underwent a drought treatment (water withheld for 0, 3, 7, and 12 days), after which they were re-watered for 5 days. Physiological indices were measured and a transcriptome sequencing analysis was performed to reveal drought response-related regulatory mechanisms. In comparison to the control, the drought treatment caused a significant increase in antioxidant enzyme activities, with levels rising up to seven times, and relative electrical conductivity from 14.5% to 78.4%, but the relative water content decreased from 88.3% to 23.4%; these indices recovered somewhat after the 5-day re-watering period. The RNA sequencing analysis identified 9126 differentially expressed genes (DEGs), which were primarily involved with abscisic acid responses, and mitogen-activated protein kinase signaling. These DEGs included 483 (5.29%) transcription factor genes from 53 families, including *ERF*, *MYB*, and *NAC*. A co-expression network analysis was conducted and three important modules were analyzed to identify hub genes, one of which (*AtruNAC36*) was examined to clarify its function. The AtruNAC36 protein was localized to the nucleus and had a C-terminal transactivation domain. Moreover, it bounded specifically to the NACRS element. The overexpression of *AtruNAC36* in *Arabidopsis thaliana* resulted in increased drought tolerance by enhancing antioxidant enzyme activities. These findings provide important insights into the transcriptional regulation mediating the *A. truncatum* response to drought. Furthermore, *AtruNAC36* may be relevant for breeding forest trees resistant to drought stress.

## 1. Introduction

As one of the most common environmental factors, drought stress severely affects seedling establishment as well as plant growth and productivity [1]. Plants adapt to drought condition through morphophysiological changes, such as stomatal closure to minimize water loss, root elongation (i.e., increasing the root-to-shoot ratio) to enhance water absorption, and cell wall modifications that promote water transport [1,2]. Additionally, metabolic processes are reorganized and the antioxidant system is activated to eliminate the excess reactive oxygen species (ROS) resulting from an exposure to drought stress. During these processes, the expression of drought stress-related genes is regulated to enhance drought stress responses at the morphological and physiological levels [3,4]. Clarifying the expression patterns of genes involved in drought responses is essential for characterizing the mechanism underlying the drought resistance of plants.

Many drought stress-inducible genes and signaling pathways have recently been identified on the basis of RNA sequencing (RNA-seq) analyses. For example, 5164 differentially expressed genes (DEGs) between two sea buckthorn subspecies under drought conditions were detected, including six hub genes involved in the abscisic acid (ABA) signaling pathway [5]. In *Pinus halepensis* Miller, 6035 DEGs mainly associated with ROS scavenging as well as fatty acid and cell wall biosynthesis were identified following a drought treatment, while the transcription of retrotransposons was observed to increase significantly after trees recovered from drought stress [6]. In *Aegilops tauschii,* 6969 DEGs primarily involved in starch and sucrose metabolism as well as mitogen-activated protein kinase (MAPK) signaling were identified in response to drought stress [7]. An examination of Norway spruce trees under drought conditions revealed 5835 and 1304 DEGs in the roots and needles [8], respectively. In *Salix psammophila*, 8172 DEGs that were previously detected in drought-stressed roots contribute to transcriptional regulation and stress responses, with two hub genes encoding positive regulators of drought resistance [9]. Therefore, key drought-related genes may be efficiently identified by performing sequencing and bioinformatic analyses.

The NAC (NAM, ATAF1/2, and CUC2) family is a plant-specific transcription factor (TF) family that affects several biological processes, including secondary wall formation, shoot apical meristem development, and responses to environmental stimuli [10,11,12]. The NAC TFs bind directly to the NAC recognition site (NACRS) [CGT(G/A)] in the promoter of the downstream target genes to regulate transcription [13]. To date, there has been substantial research conducted to functionally characterize *NAC* genes in terms of their contribution to stress tolerance. In *Arabidopsis thaliana*, the expression of *ANAC019* is triggered in response to dehydration and salinity. The encoded protein helps mediate abiotic stress defense responses by modulating jasmonate and ABA signaling [14,15]. The constitutive overexpression of *MdNAC1* from apple (*Malus hupehensis*) significantly enhances drought tolerance by increasing antioxidant enzyme activities [16]. The *MdNAC047* expression level increases significantly following an exposure to salt stress; the constitutive overexpression of this gene in *A. thaliana* and apple promotes *MdERF3* expression and increases salt tolerance [17]. The overexpression of poplar *NAC13* enhances the salt tolerance of transgenic tobacco and poplar [18,19]. In cotton, *GhirNAC2* expression can improve drought tolerance by mediating ABA biosynthesis and stomatal closure [20]. These studies reflect the importance of NAC TFs for abiotic stress tolerance.

*Acer truncatum* B. is an important oil-producing woody plant. Its tissues/organs are valued because of their potential uses [21]. For example, the seeds and leaves are a rich source of nervonic acid, which has positive effects on cranial nerves and may be incorporated into health care products or medicines [22,23]. Because of the changing leaf colors and desirable shape of *A. truncatum* trees, they are often used for landscaping [24]. Moreover, *A. truncatum* is highly tolerant to drought conditions and can grow in arid and semi-arid regions. In recent times, several researches has been conducted to analyze the physiological effects of *A. truncatum* under drought stress. For example, *A. truncatum* has been reported to be able to endure drought stress through delayed dehydration at high water potentials, and its water regulation approach is generally conservative water [25]. Chronic drought stress has a mitigating effect on the negative impacts of ozone (O_3_) on growth and physiology in *A. truncatum* [26]. The leaf size, photosynthetic rate, and stomatal conductance are reduced under drought and O_3_ stress in *A. truncatum* [26]. The similar phenotype under drought stress are detected in *A. rubrum* and *A. pictum* which are related species of *A. truncatum* [27]. However, the systematic study of gene expression in *A. truncatum* under drought stress is still lacking, and the molecular mechanism underlying the drought resistance of *A. truncatum* remains unknown. We hypothesized that *A. truncatum* was able to endure drought stress by activating certain responses and some key genes that were essential for its drought tolerance.

To verified our hypotheses, physiological and transcriptome sequencing analyses of drought-stressed *A. truncatum* samples were conducted to reveal the regulatory mechanisms associated with drought tolerance. In addition, co-expression networks for *A. truncatum* under drought conditions were constructed to screen for hub genes, one of which (*AtruNAC36*) was functionally characterized to clarify its contribution to drought tolerance. This is the first exploration of transcriptome of *A. truncatum* under drought stress. The findings of this study provide researchers with useful information regarding the transcriptional regulatory mechanism mediating the *A. truncatum* drought response. Notably, a candidate gene relevant for improving the drought resistance of forest tree species was identified.

## 2. Materials and Methods

### 2.1. Plant Materials and Drought Treatment

*Acer truncatum* (Act-E) materials were acquired from Jiulong Mountain (E 115°59′–116°06′, N 39°54′–39°57′) in Beijing. Act-E was an excellent tree that were selected from Jiulong Mountain. The tree grew rapidly, with a height of 18.6 m and a diameter of 0.73 m at breast height in 20-year-old. The trunk was vertical, and the foliage was dense, with few infestations or illnesses. The twigs from Act-E were divided into 15-cm segments (one leaf per segment) and then added to plastic pots containing a turfy soil/perlite/vermiculite mixture with soil moisture at 75% in a greenhouse at the Experimental Center of Forestry in North China. After 1 year, vigorously growing and uniformly sized seedlings were selected for the drought treatment. These seedlings were stopping irrigation at water for 12 days to simulate a natural drought event and re-watered for 5 days (RW) [28]. On 0 (control), 3, 7, 12 days under drought stress and RW, the second and third leaves (from the stem tip) were collected from five seedlings at each time-point and frozen immediately using liquid nitrogen and stored at −80 °C for RNA extraction. For each time point, three independent biological replicates were conducted. Each biological replicate contained five individual seedlings. In addition, the fourth and fifth leaves were collected to examine physiological indices, including relative electrical conductivity (REC), relative water content (RWC), peroxidase (POD) activity, superoxide dismutase (SOD) activity, and catalase (CAT) activity. REC and RWC were measured according to the Hu’ study [28]. POD, SOD, and CAT activities were detected using reagent kits from Solarbio Company (Beijing, China) according to the manufacturer’s protocols. Three biological replicates and five technical replicates in the biochemical analysis were conducted.

### 2.2. RNA Extraction and RNA-Seq Analysis

Total RNA of 15 leaf samples (water withheld for 0, 3, 7, 12 days, and re-watered for 5 days, three independent biological replicates per time-point) was extracted using the RNA prep Pure Plant Kit from Tiangen Company (Beijing, China) according to the manufacturer’s instructions for RNA-seq. The RNA integrity and purity were determined using the Agilent 2100 system (Agilent Technologies, Palo Alto, CA, USA) and the NanoDrop 2000 system (Thermo Scientific, Waltham, MA, USA). The high-quality RNA (RNA integrity number ≥ 8.0, optical density ≥ 2.0, and concentration ≥ 100 ng/µL) was used to construct libraries that were sequenced on the Illumina HiSeq 2500 platform (2 × 125-bp paired-end reads) at BMKCloud Technologies Corporation (Shenzhen, China). The raw RNA-seq data for *A. truncatum* under drought conditions were deposited in the National Genomics Data Center database (PRJCA013039) (https://bigd.big.ac.cn, accessed on 13 November 2022).

### 2.3. Sequence Data Analysis and Functional Annotation

After eliminating the low-quality reads, adapters, and reads with poly-N sequences, the clean data were mapped to the *A. truncatum* genome sequence using the Bowtie2 software [29]. Gene expression levels were measured according to the fragments per kilobase of transcript per million mapped fragments (FPKM) value. Princomp was used to perform a principal component analysis (PCA). The DEGs between groups were identified using EdgeR and the following criteria: ratio of gene expression levels in two samples ≥ 2 and false discovery rate ≤ 0.05.

The GOseq R package and KOBAS software were used for the Gene Ontology (GO) enrichment analysis [30] and the Kyoto Encyclopedia of Genes and Genomes (KEGG) pathway analysis [31].

### 2.4. Co-Expression Network Analysis

A co-expression network analysis was performed using the weighted gene co-expression network analysis (WGCNA) package in R. The WGCNA edge weights > 0.30 were considered valid. Connectivity (degree) was calculated as the number of all node edges, and genes with a degree exceeding 90 in the network were defined as hub genes. The Cytoscape 3.7.0 software was used for visualizing the co-expression network.

### 2.5. Transcriptional Activation Assay

The *AtruNAC36* (*Atru.chr5.3491*) coding region was amplified via a polymerase chain reaction using specific primers (Table S1). The full-length *AtruNAC36* sequence, the sequences encoding the N-terminal (amino acids 1–153) and C-terminal (amino acids 154–298) of AtruNAC36 were ligated into separate pGBKT7 vectors (Clontech, Palo Alto, CA, USA). The recombinant plasmids were inserted into separate Y2HGold yeast cells, which were cultured for 3 days on solid SD medium lacking tryptophan (SD/−Trp) and solid SD medium lacking Trp, histidine (His), and adenine (Ade), but supplemented with X-α-Gal (SD/−3/X-α-Gal). The pGBKT7 vector was used as negative control. The growth status of the transformed yeast cells and the blue/white colony assay results were used to determine transcriptional activation. The PCR primers are listed in Table S1.

### 2.6. Yeast One-Hybrid (Y1H) Assay

Three tandem copies of the NACRS sequence (CGTA; R) and the mutated sequences RM1 (CGCC) and RM2 (AATA) were cloned into separate pHIS2 vectors form three bait reporter vectors. The *AtruNAC36* coding sequence was inserted into the pGADT7-Rec2 vector for a pGADT7-Rec2-AtruNAC36 fusion vector. Different combinations of the recombinant pGADT7-Rec2 and bait reporter vectors s were co-expressed in Y187 yeast cells, which were cultured on solid SD/−leucine (Leu)/−Trp medium (DDO). The transformed yeast cells form DDO medium were proliferated and diluted 10, 100, 1000 times with autoclaved double-distilled water and then dripped on solid SD/−Leu/−Trp/−His medium supplemented with 3-amino-1,2,4-triazole (3-AT) (TDO + 50 mM 3-AT). The positive control yeast cells were transformed with pHIS2-p53 and pGADT7-Rec2-p53, whereas the negative control yeast cells were transformed with pHIS2-p53 and pGADT7-Rec2-AtruNAC36.

### 2.7. Analysis of the Drought Tolerance of AtruNAC36-Overexpressing Transgenic A. thaliana

The *AtruNAC36* coding sequence was inserted into the pCAMBIA1302 vector using ClonExpress II One Step Cloning Kit (Vazyme, Nanjing, China) to construct the *35S::AtruNAC36* recombinant plasmid, which was used to transform *A. thaliana* plants according to the floral dip method [32]. After screened using hygromycin, more than 30 *AtruNAC36*-overexpressing transgenic lines were obtained, of which two homozygous *AtruNAC36*-overexpressing transgenic lines with high relative *AtruNAC36* expression levels were selected for the drought treatment. Two developmental stages of *AtruNAC36*-overexpressing transgenic seedling were chosen for drought stress tolerance analysis. The root length, fresh weight, survival rate, REC and RWC were measured and the 3, 3′-diaminobenzidine (DAB) and nitroblue tetrazolium (NBT) staining were performed as previously described [33].

## 3. Results

### 3.1. Physiological Responses of A. truncatum to Drought Stress

**Figure 1:**
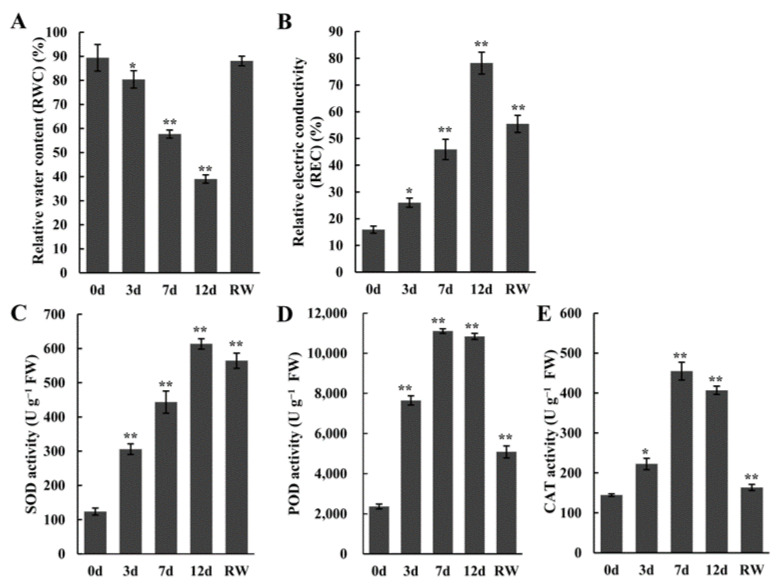
Analysis of *A. truncatum* physiological indices. (**A**) Relative water content (RWC), (**B**) relative electrical conductivity (REC), (**C**) superoxide dismutase (SOD) activity, (**D**) peroxidase (POD) activity, and (**E**) catalase (CAT) activity in *A*. *truncatum* after withholding water for 0, 3, 7, and 12 days and then re-watering for 5 days (RW). Error bars represent standard deviations. Asterisks represent significant differences according to Student’s *t*-test (* *p* < 0.05; ** *p* < 0.01).

To explore the physiological responses of *A. truncatum* to drought stress, *A. truncatum* seedlings were withheld water for a number of days (i.e., 0, 3, 7, and 12 days) to simulate natural drought stress and then re-watered for 5 days (i.e., RW). The physiological indices of the seedlings were examined at these five time-points. The leaves gradually wilted as the duration of the simulated drought period increased (Figure S1), but after the seedlings were re-watered, the leaves recovered and appeared normal (Figure S1). The examination of specific physiological indices during the drought treatment period (0–12 days) revealed that RWC decreased gradually from 88.3% to 23.4% in response to drought stress, whereas REC increased significantly from 14.5% to 78.4%, reflecting the drought-induced damages to the seedlings. Both RWC and REC recovered after watering was resumed for 5 days. In addition, the SOD, POD, and CAT activities were increased as the drought treatment progressed, but then decreased after seedlings were re-watered (Figure 1C–E).

### 3.2. Identification of DEGs in A. truncatum under Drought Conditions

To clarify the molecular mechanism regulating the *A. truncatum* response to drought stress, an RNA-seq analysis was performed to reveal the drought-induced changes to gene expression. Paired-end read lengths of 125 bp were applied. A total of 98.08 Gb clean reads were obtained for the 15 samples (three replicates per time-point) with a Q30 score of at least 91.30%. Additionally, 89.53–92.16% of the reads were mapped to the *A. truncatum* genome. The detail information of RNA-seq data of *A. truncate* was listed in Table S2.

A PCA was performed to evaluate the uniformity of the data for the three biological replicates and the samples at five time-points. The first two principal components (PC1 and PC2) explained 87.83% of the total variation (Figure S2). The clustering of the three biological replicates for each time-point was indicative of the high repeatability of the results. The samples collected at the five time-points (0 d, 3 d,) were obviously separated. The distance separating samples was smallest between the day 0 and day 3 samples, followed by the day 0 and RW samples. In contrast, there were relatively large distances between the day 3 and day 7 samples as well as between the day 7 and day 12 samples and between the day 12 and RW samples (Figure S2).

**Figure 2:**
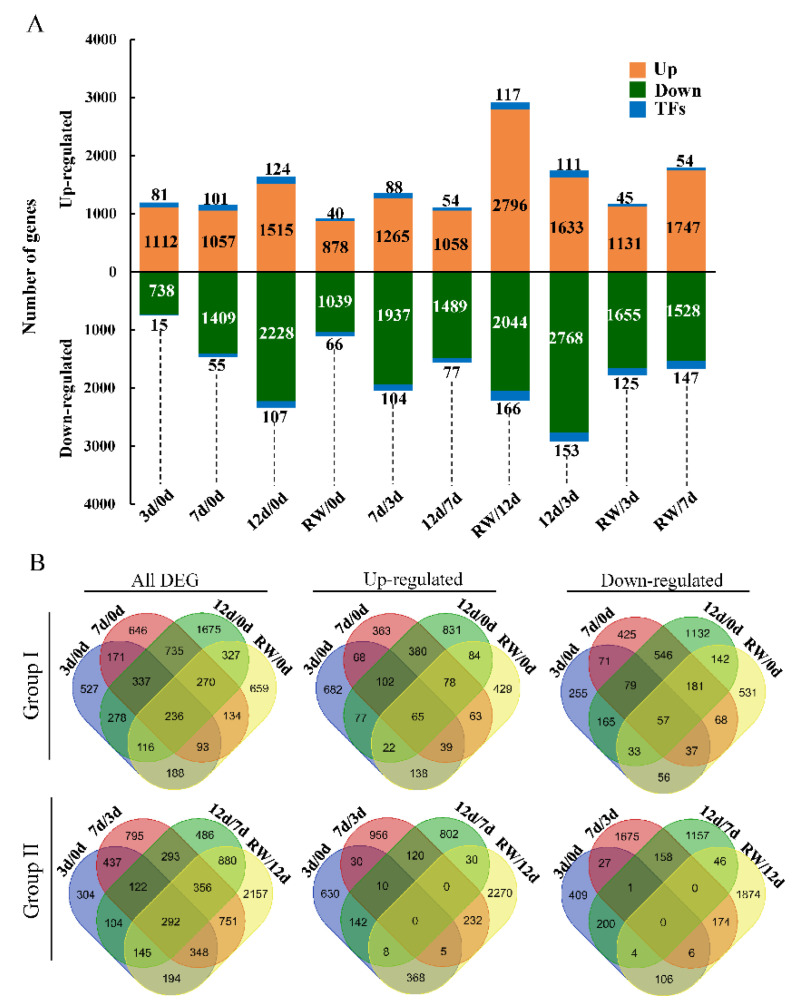
Analysis of the DEGs in *A. truncatum* under drought conditions. (**A**) Bar graph presenting the number of up-regulated DEGs, down-regulated DEGs, and transcription factor genes in each comparison. (**B**) Venn diagram indicating the number of common or unique DEGs in the Group I comparisons of four treatment time-points (3 d/0 d, 7 d/0 d, 12 d/0 d, and RW/0 d) and the Group II time-course comparisons (3 d/0 d, 7 d/3 d, 12 d/7 d, and RW/12 d).

A total of 9126 DEGs were detected among the five time-points (Figure 2A and Table S3). The 11 comparisons that were completed were divided into two groups. More specifically, Group I comprised the comparisons between the samples on day 0 and the samples at the other time-points (day 3, day 7, day 12, and RW samples), whereas Group II consisted of the time-course comparisons (3 d/0 d) (day 3 vs. day 0), 7 d/3 d, 12 d/7 d, and RW/7 d and other comparisons 12 d/3 d, RW/3 d, and RW/7 d. In Group I, the day 12 d/0 d comparison had the most DEGs (3974), of which 1639 and 2335 were up-regulated and down-regulated genes, respectively. The fewest DEGs (1946) were detected for the 3 d/0 d comparison, which was consistent with the close distance between the day 0 and day 3 samples. In Group II, the RW/12 d comparison had the most DEGs (5123), which included 2913 up-regulated genes and 2210 down-regulated genes. Some of the DEGs were common among comparisons (Figure 2). For example, 337 DEGs were detected in the 3 d/0 d, 7 d/0 d, and 12 d/0 d comparisons.

Transcription factors are crucial transcriptional regulators of plant responses to drought stress. A total of 483 TF genes from 53 gene families were identified among the detected DEGs (Table S4). The TF genes were mainly from the *ERF* family (47 genes), followed by the *MYB* (46 genes) and *NAC* (38 genes) families. Their expression levels were affected by drought stress. For example, the 12 d/0 d comparison revealed 124 and 107 TF genes with up-regulated and down-regulated expression levels, respectively (Figure 2).

To assess the reliability of the RNA-seq data, the following eight genes were selected for a quantitative real-time (qRT)-PCR analysis to determine their expression levels under drought conditions: *Atru.chr5.3491* (*AtruNAC36*), *Atru.chr7.1057* (MAPK family member), *Atru.chr9.1574* (MYB family member), *Atru.chr1.144* (protein phosphatase 2C family member), *Atru.chr1.2580* (WRKY family member), *Atru.chr1.3172* (ABC-2 type transporter), *Atru.chr13.381* (ERF family member), and *Atru.chr8.1096* (late embryogenesis abundant family member). For all eight genes, similar expression trends were revealed by the qRT-PCR and RNA-seq analyses (Figure S3), indicative of the reliability of the transcriptome data.

### 3.3. GO and KEGG Enrichment Analyses of DEGs

**Figure 3:**
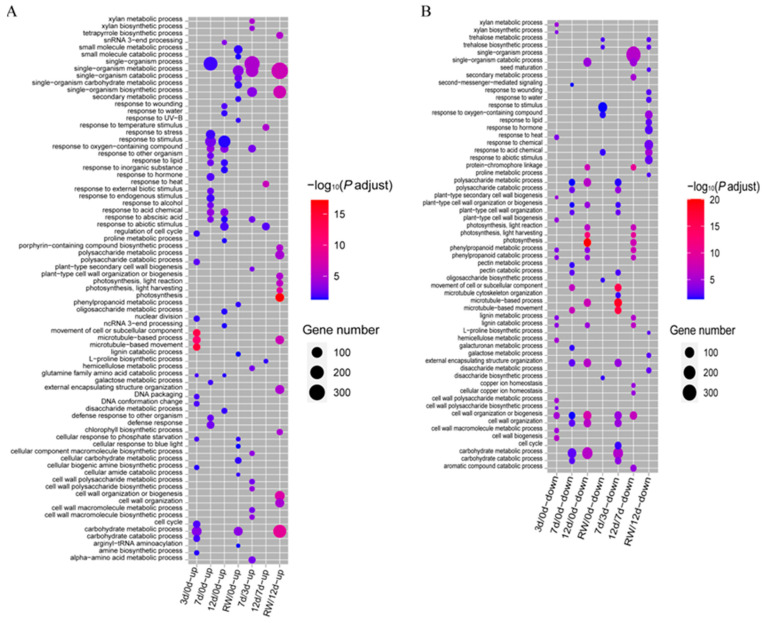
GO enrichment analysis of the DEGs in *A. truncatum* under drought conditions. (**A**,**B**) GO enrichment analysis of the up-regulated (**A**) and down-regulated (**B**) genes.

The DEG were functionally characterized according to a GO enrichment analysis (Figure 3). In the early drought stage (3 days), the enriched GO terms among the up-regulated genes were “carbohydrate metabolic process”, “carbohydrate catabolic process”, “microtubule-based process”, and “microtubule-based movement” (Figure 3A, Table S5). In the middle and later drought stages, the enriched GO term among the prominent up-regulated genes in the 7 d/0 d and 12 d/0 d comparisons was “stress related processes” (e.g., response to wounding, stress, water, and abiotic stimuli). Some of the up-regulated genes were also annotated with the “response to abscisic acid” and “response to lipid” GO terms. For the RW/12 d comparison, “stress related processes” and “photosynthesis” were the enriched GO terms assigned to the down-regulated and up-regulated genes, respectively (Figure 3), suggesting the 5-day re-watering period enabled the seedlings to recover from drought stress.

**Figure 4:**
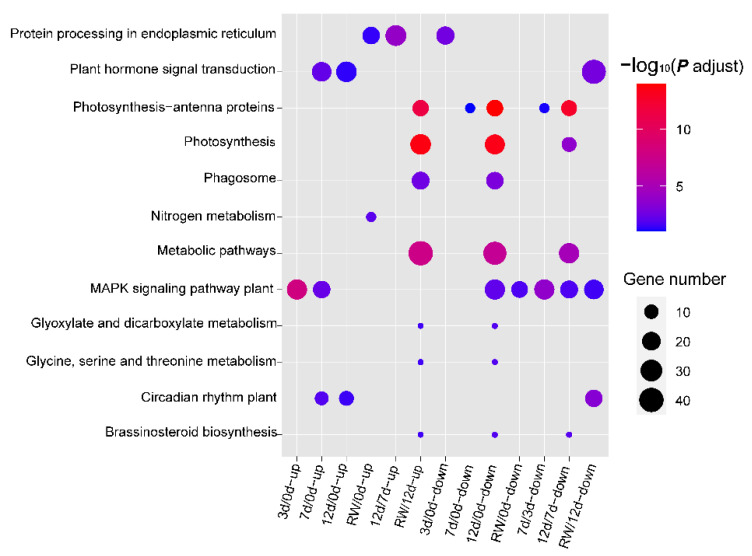
KEGG enrichment analysis of the DEGs in *A. truncatum* under drought conditions.

The KEGG analysis, which was completed to further explore the metabolic changes in *A. truncatum* induced by drought conditions, identified 12 enriched pathways (Figure 4, Table S6). The MAPK signaling pathway was a common enriched KEGG pathway among the DEGs detected in most comparisons. Both “photosynthesis–antenna proteins” and “photosynthesis” were enriched KEGG pathways related to the DEGs detected in the RW/12 d, 12 d/0 d, and 12 d/7 d comparisons. Moreover, according to the enriched KEGG pathways (e.g., “plant hormone signal transduction” and “brassinosteroid biosynthesis”), the DEGs revealed by some comparisons were associated with hormone-related processes (Figure 4).

### 3.4. Cluster Analysis of DEGs

**Figure 5:**
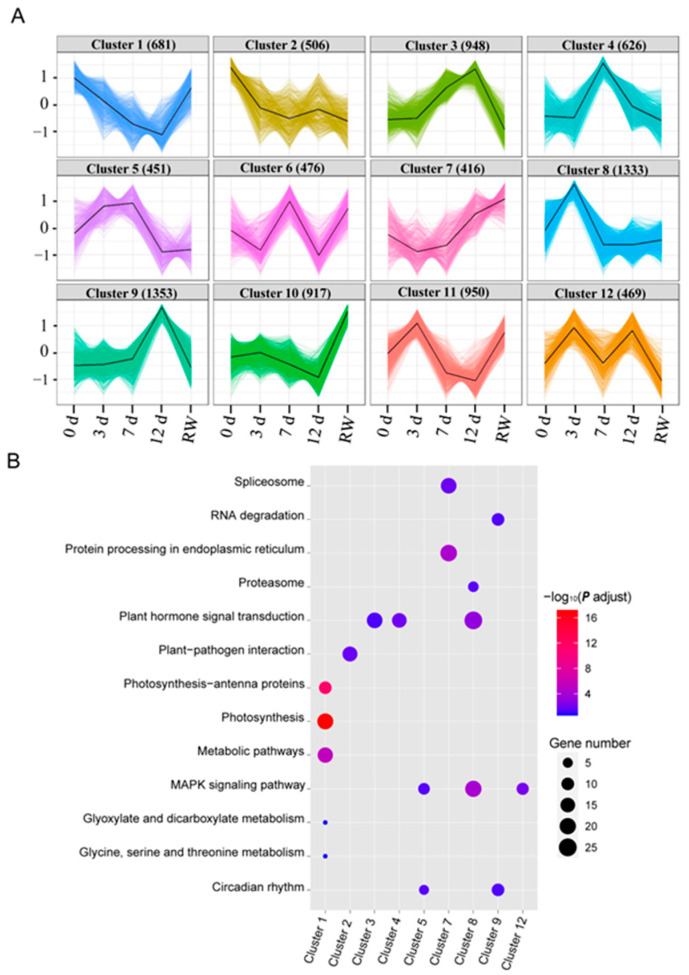
Cluster analysis of the DEGs in *A. truncatum* under drought conditions and KEGG enrichment analysis. (**A**) Expression patterns for the genes in 12 clusters. (**B**) KEGG enrichment analysis of the DEG clusters.

The dynamic molecular responses of *A. truncatum* exposed to drought stress were further elucidated by classifying the DEGs on the basis of their expression trends. The 9126 DEGs were divided into 12 clusters (Figure 5, Table S7). The 681 genes whose expression levels were initially down-regulated in response to drought stress, but then rebounded during the recovery period, were grouped in Cluster 1; these genes were mainly associated with photosynthesis-related processes (e.g., photosynthesis, light harvesting, and the light reaction). Cluster 8 comprised 1333 genes with significantly up-regulated expression levels on day 3 of the drought treatment; the enriched KEGG pathways among these genes were “MAPK signaling pathway”, “plant hormone signal transduction”, and “proteasome”. The expression of the 626 genes in Cluster 4 was substantially induced on day 7 of the drought treatment; “plant hormone signal transduction” was the enriched KEGG pathway assigned to these genes. The expression of the genes in Clusters 3 and 9 peaked on day 12 of the drought treatment; the main KEGG pathways associated with these genes were “plant hormone signal transduction”, “RNA degradation”, and “circadian rhythm” (Table S8).

### 3.5. Co-Expression Network Analysis

**Figure 6:**
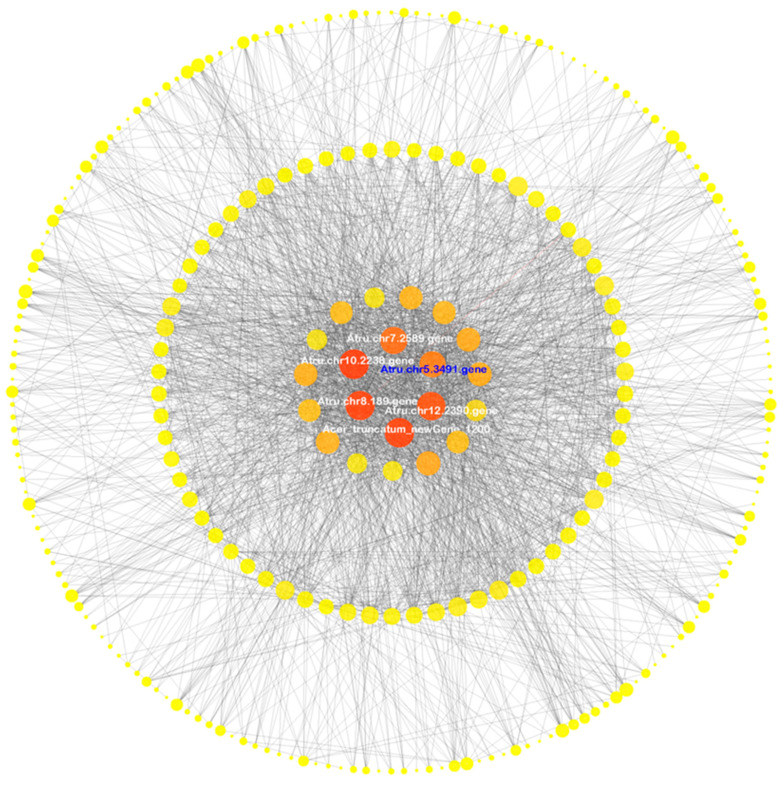
Co-expression network for the yellow module. The nodes represent the DEGs in the yellow module, whereas the lines represent the correlation between two DEGs. Connectivity was defined as the number of all node edges. Node size and color intensity reflect the connectivity between genes. Small and pale yellow nodes indicate low connectivity, whereas large and dark yellow nodes indicate high connectivity. The genes in the innermost circle are considered to be the hub genes in this module. The *NAC36* (*Atru.chr5.3491*) gene is marked in blue and was selected for the functional verification.

To identify the key DEGs involved in the *A. truncatum* drought response, a WGCNA was conducted, which resulted in 12 distinct modules that were differentiated according to color in a dendrogram (Figure S4). The turquoise module was largest (2427 DEGs), followed by the blue (2325 DEGs) and yellow (747 DEGs) modules. In the co-expression network for the turquoise module, the genes encoding the gibberellin receptor AtruGID1B (*Atru.chr5.1308*), a receptor kinase (*Atru.chr6.2180*), the ethylene-responsive transcription factor AtruERF110 (*Atru.chr13.2060*), and a fatty acid biosynthesis-related protein (*Atru.chr13.1709*) had the highest connectivity values. In the co-expression network for the blue module, the highest connectivity values were obtained for the genes encoding the kinesin-like protein AtruKIN-12F (*Atru.chr2.3626*), microtubule-associated protein 3 (*Atru.chr1.633*), mitotic-specific cyclin AtruS13-6 (*Atru.chr1.1390*), and cellulose synthase-like protein D5 (*Atru.chr5.1070*). In the co-expression network for the yellow module, the genes encoding AtruNAC36 (*Atru.chr5.3491*), an LRR receptor-like serine/threonine-protein kinase (*Acer_truncatum_newGene_11938*), and AtruWRKY50 (*Atru.chr7.1891*) had the highest connectivity values. Thus, we speculated that the genes in the turquoise, blue, and yellow modules may mediate the *A. truncatum* response to drought stress. The co-expression networks for these three modules were visualized (Figure 6, Figure S5 and Figure S6, Table S9).

### 3.6. Isolation and Characterization of AtruNAC36

**Figure 7:**
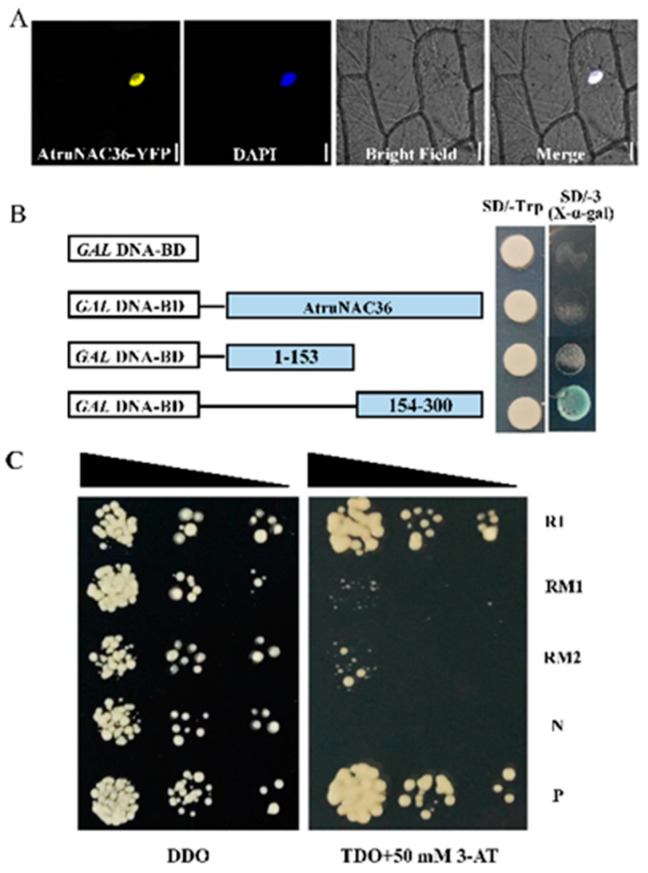
Characterization of AtruNAC36 in *A. truncatum*. (**A**) Subcellular localization of AtruNAC36. The fluorescence of YFP (*35S::YFP-AtruNAC36*) was detected in onion epidermal cells. (**B**) Transcriptional activation analysis of AtruNAC36 using a yeast system. The structures of the full-length AtruNAC36 as well as the N-terminal and C-terminal are presented. All constructs were expressed in yeast cells, which were grown on SD/−Trp medium and SD/−Trp/−His/−Ade medium containing 50 mM 3-AT. (**C**) AtruNAC36 was revealed to bind the NACRS element. More specifically, the ability of AtruNAC36 to recognize NACRS (R) and the mutated sequences (RM1 and RM2) was assessed. Serially diluted (1, 10^−1^, and 10^−2^) transformants were grown on DDO medium and TDO medium containing 3-AT.

Among the hub genes, *AtruNAC36* had a high connectivity value in the co-expression network for the yellow module. Additionally, its expression levels increased significantly on days 3, 7, and 12 of the drought treatment, indicative of the importance of the encoded protein throughout the exposure to drought stress. Thus, *AtruNAC36* was functionally characterized. The protein structural analysis suggested AtruNAC36 contains a NAC domain (amino acids 6-152). The sequence alignment detected five subdomains in AtruNAC36 (Figure S7). To examine the subcellular localization of AtruNAC36, *35S::YFP*-*AtruNAC36* was constructed to produce AtruNAC36 with an N-terminal YFP in onion epidermal cells. The fluorescence of YFP was detected in the nucleus, indicating that AtruNAC36 is a nuclear protein (Figure 7A).

In addition, AtruNAC36 was analyzed in terms of transcriptional activation. The *AtruNAC36* coding sequence and the sequences encoding the N-terminal and C-terminal of the protein were inserted into pGBKT7 vectors, which were then used to transform yeast cells. All transformants grew on the SD/−Trp medium, but only the yeast cells harboring the C-terminal of AtruNAC36 were able to grow on the SD/−Trp/−His/−Ade medium (i.e., blue colonies) (Figure 7B). Hence, the C-terminal of AtruNAC36 appears to be sufficient for transcriptional activation.

To determine whether AtruNAC36 can bind to NACRS, the tandemly repeated NACRS sequence (R) and the mutated sequences (RM1 and RM2) were used for Y1H assays. All yeast cells grew on the DDO (SD/−Leu/−Trp) medium (Figure 7B). In contrast, the yeast cells carrying pGAD-AtruNAC36/pHIS2-NACRS (R) and the positive control (P) grew normally on the TDO (SD/−Leu/−Trp/−His) medium supplemented with 50 mM 3-AT, but the growth of the cells with the mutated sequences (RM1 and RM2) and the negative control (N) was inhibited (Figure 7C). These results confirmed that AtruNAC36 can bind specifically to NACRS.

### 3.7. Overexpression of AtruNAC36 Enhanced Drought Tolerance

**Figure 8:**
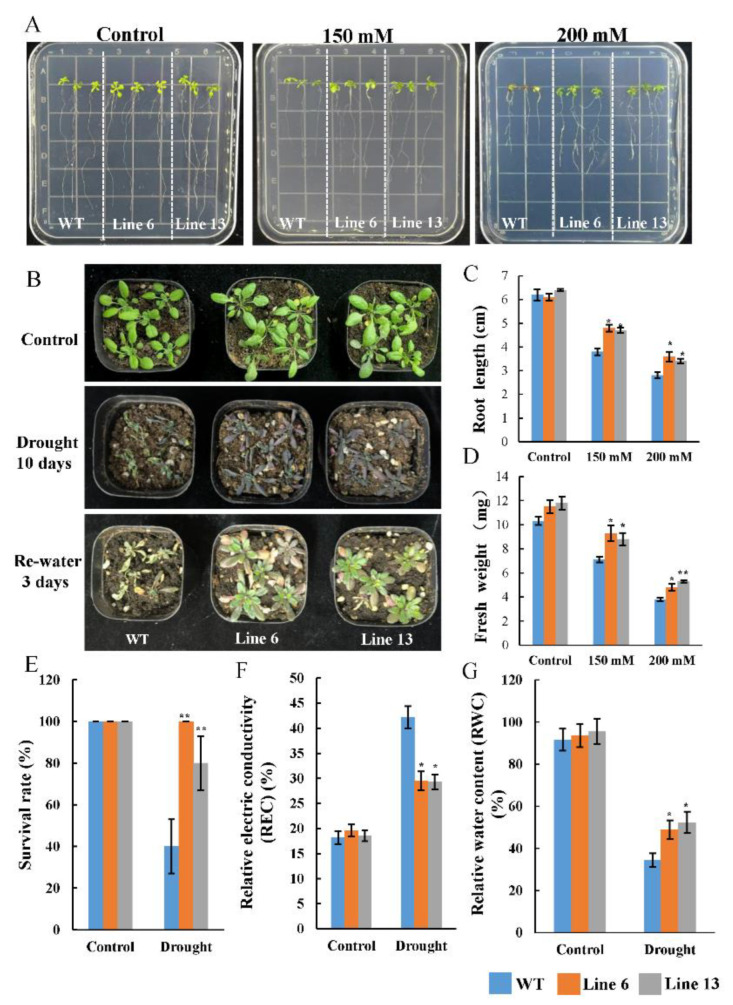
*AtruNAC36* overexpression increased the drought tolerance of *Arabidopsis thaliana*. (**A**) Phenotype of *AtruNAC36*-overexpressing and WT seedlings in the normal medium and mannitol medium (150 and 200 mM mannitol). (**B**) Phenotypic characteristics of *AtruNAC36*-overexpressing and WT plants in soil under normal-watering conditions, drought conditions for 10 days, or after re-watering for 3 days. (**C**,**D**) Root length (**C**) and fresh weight (**D**) of the *AtruNAC36*-overexpressing and WT seedlings in the normal medium and mannitol medium (150 and 200 mM mannitol). (**E**) Survival rate after re-watering for 3 days. (**F**,**G**) Relative electrical conductivity (REC) (**F**) and relative water content (RWC) (**G**) of the *AtruNAC36*-overexpressing and WT. Plants.Significance test was conducted using Student’s *t*-test (* *p* < 0.05 and ** *p* < 0.01).

To elucidate the *AtruNAC36* function, *AtruNAC36*-overexpressing transgenic *A. thaliana* lines were generated, of which lines 6 and 13 had high *AtruNAC36* expression levels and were selected for an assessment of drought tolerance (Figure S5). Under normal conditions, there were no significant differences in the root length, fresh weight, REC, and RWC between the wild-type (WT) and transgenic seedlings (Figure 8). To further clarify the role of AtruNAC36 during the *A. truncatum* response to drought stress, seedlings were grown on MS medium for 1 week and then transferred to mannitol medium to simulate drought conditions. The growth of the WT and *AtruNAC36*-overexpressing transgenic seedlings was significantly suppressed after 1 week in the mannitol medium. However, the WT seedlings had shorter roots and a lower fresh weight than the *AtruNAC36*-overexpressing seedlings. More specifically, the average root lengths for the WT and *AtruNAC36*-overexpressing seedlings in the 150 mM mannitol medium were 3.8 and 4.7 cm, respectively (Figure 8A,C). Additionally, drought tolerance was evaluated using adult plants. After withholding water for 10 days, the transgenic plants had fewer wilted and curled rosette leaves than the WT plants. Consistent with the observed phenotypes, the transgenic plants had a significantly higher RWC, but a lower REC, than the WT plants under drought conditions. Furthermore, after the re-watering period, the transgenic plants had a higher survival rate than the WT plants (i.e., better recovery from drought stress). Accordingly, the overexpression of *AtruNAC36* enhanced drought tolerance.

### 3.8. Overexpression of AtruNAC36 Increased ROS-Scavenging Efficiency

**Figure 9:**
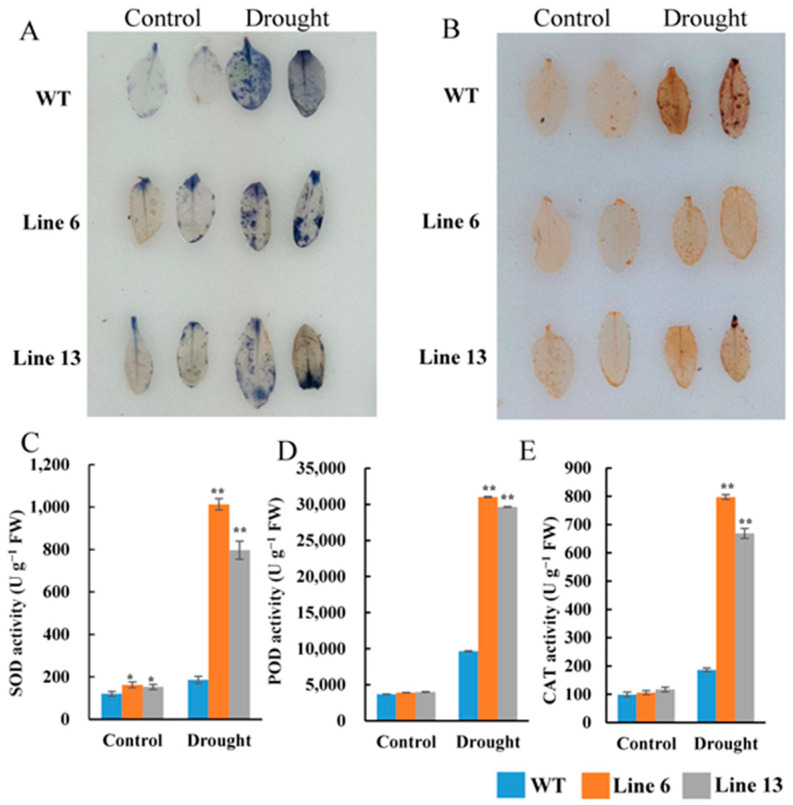
*AtruNAC36* overexpression enhanced the activity of antioxidant enzymes in *Arabidopsis thaliana*. (**A**,**B**) DAB (**A**) and NBT (**B**) staining analyses were performed using the rosette leaves from the WT and *AtruNAC36*-overexpressing seedlings under normal and drought conditions. (**C**–**E**) POD, SOD, and CAT activities in the WT and *AtruNAC36*-overexpressing seedlings under normal and drought conditions. Error bars represent standard deviations. Asterisks represent significant differences according to Student’s *t*-test (* *p* < 0.05; ** *p* < 0.01).

Drought stress leads to the excessive generation of ROS. The associated inhibitory effects on plant growth may be mitigated by ROS-scavenging antioxidant enzymes. In this study, the H_2_O_2_ and O_2_^−^ contents in the leaves of WT and *AtruNAC36*-overexpressing plants were analyzed via NBT and DAB staining. The number of colored regions and the intensity of the staining were greater for the WT leaves than for the *AtruNAC36*-overexpressing leaves following the NBT and DAB staining analyses of the drought-treated seedlings (Figure 9). These results indicated that the H_2_O_2_ and O_2_^−^ contents were lower in the *AtruNAC36*-overexpressing plants than in the WT plants exposed to drought stress.

Furthermore, antioxidant enzyme activities were measured. Under normal conditions, *AtruNAC36*-overexpressing plants had higher SOD activities than the WT plants, whereas there were no differences in the POD and CAT activities. Under drought conditions, the SOD, POD, and CAT activities increased significantly in the transgenic plants relative to the corresponding levels in the WT plants. Hence, the overexpression of *AtruNAC36* increased the antioxidant enzyme activities.

## 4. Discussion

Drought stress severely inhibits plant development and induces a series of physiological changes [6]. In the current study on *A. truncatum*, the leaf RWC gradually decreased and the REC increased (Figure 1) during the 12-day drought treatment period, suggesting that the extent of the cell membrane damage increased as the exposure to drought stress was prolonged. Drought stress also leads to oxidative damage. Accordingly, the activities of antioxidant enzymes (e.g., POD, SOD, and CAT) are induced to eliminate the excessive amounts of ROS. In *A. truncatum*, the SOD, POD, and CAT activities increased under drought conditions (day 0 to day 12). The enhanced antioxidant enzyme activities indicated that the *A. truncatum* response to drought stress may involve the modulation of ROS homeostasis, thereby limiting cellular damage.

The regulated transcription of stress-related genes is an important molecular mechanism underlying adaptive responses. In the present study, 9126 DEGs were identified. The expression levels of the genes involved in “photosynthesis” were down-regulated under drought conditions, but recovered after the re-watering period according to the RW/12 d comparison. These findings reflected the adverse effects of drought stress on photosynthetic activities. The KEGG analysis revealed that many of the DEGs detected in the current study are involved in the MAPK signaling pathway. Earlier research confirmed that MAPK signal transduction plays a vital role in eukaryotic responses to extracellular stimuli [34]. Several MAPK family members have been isolated and functionally characterized in multiple plants. For example, in maize, *ZmMAPK1* expression is induced by drought stress; the overexpression of this gene in transgenic plants leads to drought tolerance [35]. The expression of *SlMAPK1* in tomato is also induced under drought conditions; the constitutive overexpression of *SlMAPK1* increases drought tolerance through enhanced antioxidant enzyme activities [36]. In plants, MAPK cascade is responsible for controlling the antioxidant defense system and osmotic regulation system [37]. The antioxidant activities were increased in *A*. *truncatum* under drought stress which indicated that MAPK signal transduction pathway was implicated in drought stress in *A*. *truncatum*, and it is essential to look into the functions of the DEGs that enriched in MAPK signaling pathway in greater detail.

In addition, one of the enriched GO terms assigned to some of the identified DEGs was “response to abscisic acid”. ABA is a phytohormone that functions as a chemical signal that influences plant development and responses to adverse conditions. When plants are exposed to drought stress, endogenous ABA levels increase, while physiological processes (e.g., stomatal closure) are activated and the expression of stress-responsive genes is induced to enable plants to adapt to drought conditions [38]. ABA also regulated the production of ROS, and H_2_O_2_ causes the phosphorylation of MAPK, which is a factor in multiple signal transduction pathways that control gene expression downstream [39]. In the 7 d/0 d and 12 d/0 d comparisons, respectively, 16 and 17 DEGs were found to be upregulated, and these DEGs were enriched in ABA response process. These results imply the regulation of ABA signaling may be critical for the *A*. *truncatum* response to drought stress. Further research is needed to determine if the above DEGs are involved in ROS production and MAPK signaling pathways under drought stress.

Transcription factors regulate various biological processes. Many TF families, such as ERF, MYB, WRKY, and NAC, comprise important regulators of plant responses to abiotic stress. In the current study, 483 TF genes from 53 families (e.g., *ERF*, *MYB*, and *NAC*) were detected as DEGs. The *ERF* genes encode plant-specific TFs, most of which positively affect plant responses to drought stress. The overexpression of *AtERF1* and *AtERF019* can enhance the drought tolerance of *A. thaliana* [40,41]. Similar findings have been reported for the overexpression of *ZmEREBP60* [42], *OsERF115* [43], and *OsERF71* [44]. In *A*. *truncatum*, *AtruDREB28* was induced by drought stress and play positive role in drought stress resistance in our previous study [45]. Additionally, *MYB* genes also contribute to plant defenses against drought stress. Examples include *PbrMYB21* in *Pyrus betulaefolia* [46], *MbMYB4* in *Malus baccata* [47], and *IbMYB48* in sweet potato [48]. On the basis of their expression patterns in *A*. *truncatum* and the functions of their orthologs in other species, these induced TF genes may be useful for improving *A*. *truncatum* drought tolerance.

Following our WGCNA, co-expression networks consisting of drought stress-related genes in *A*. *truncatum* were constructed and several important hub genes were identified, including *AtruNAC36*, which belongs to the *NAC* family. Several *NAC* genes have been analyzed and functionally characterized in many plants [14,15,49], but relatively little is known about *NAC* genes in *A*. *truncatum*. In the present study, sequence alignments indicated that AtruNAC36 has a diverse C-terminal domain and a conserved N-terminal domain that includes five conserved subdomains. Moreover, transactivation was observed for the AtruNAC36 C-terminal region, but not for the full-length sequence. Furthermore, in an earlier study involving a yeast self-activation detection system, the LVFY motif in the N-terminal domain of NAC was demonstrated to have repressive effects [50], which likely explains the lack of transactivation for the full-length AtruNAC36 protein. Thus, AtruNAC36 activity depends on its interaction with other proteins or structures in vivo. In addition, AtruNAC36 can specifically bind to the NACRS element, indicating that AtruNAC36 might regulate its downstream target genes by binding to NACRS in the promoter. However, future research will need to analyze the target genes and interacted proteins of AtruNAC36 in more detail to reveal its molecular mechanism.

Two developmental stages of *AtruNAC36*-overexpressing *A. thaliana* plants were examined to further clarify the AtruNAC36 function. Two-week old *AtruNAC36*-overexpressing seedlings had longer roots and a higher fresh weight than the WT seedlings in mannitol medium. The *AtruNAC36*-overexpressing adult plants had a higher survival rate and RWC, but a lower REC, than the WT adult plants. The plants overexpressing *AtruNAC36* also had significantly higher SOD, POD, and CAT activities, but lower H_2_O_2_ and O_2_^−^ contents, than the WT controls. These findings suggest that *AtruNAC36* confers drought tolerance by promoting the activity of ROS-scavenging enzymes. The functional verification of *AtruNAC36* further confirmed the reliability of our method for identifying hub genes.

## 5. Conclusions

In this study, physiological and transcriptome sequencing analyses of *A. truncatum* samples that had been exposed to drought were performed to understand the regulatory mechanisms associated with drought tolerance. Antioxidant enzyme activities were significantly induced after drought treatment, indicating *A. truncatum* might limit cellular damage through modulation of ROS homeostasis to resist drought stress. The identified DEGs were primarily involved in activating ABA responses and MAPK signaling. *AtruNAC36* was identified as one of a major hub genes from co-expression network, and the overexpression of *AtruNAC36* in *A. thaliana* could enhance the drought tolerance of transgenic plants, suggesting that the construction of co-expression network was an efficient strategy to screen key genes from a large number of DEGs. These results provide insights into the transcriptional regulatory mechanism underlying the *A. truncatum* response to drought stress. Additionally, *AtruNAC36* can serve as a candidate gene to improve the drought tolerance of forest trees through molecular breeding.

## Data Availability

The data presented in this study are available in Supplementary Materials.

## Supplementary Materials

The following supporting information can be downloaded at: https://www.mdpi.com/article/10.3390/antiox12071339/s1, Figure S1. Phenotypic characteristics of *A. truncatum* under drought conditions. Figure S2. Principal component analysis of three biological replicates and 15 samples at five time-points. Figure S3. Quantitative real-time (qRT)-PCR analysis of eight genes. Gray columns represent the FPKM values of the eight genes at five time-points (calculated using RNA-seq data for 15 samples) and red lines indicate the relative expression levels of the eight genes as determined by qRT-PCR. Transcript levels at day 0 were set to 1. Enclosed in brackets are the Pearson correlation coefficients between FPKM values from RNA-Seq and RGE values from qPCR. Figure S4. Weighted gene co-expression network analysis of the DEGs in *A. truncatum* under control and stress conditions. (A) Dendrogram of all DEGs based on the WGCNA analysis, with 12 modules defined. (B) The eigengene network shows the relationships among the 12 modules. Figure S5. Cytoscape visualization of the co-expression network in the turquoise module. Figure S6. Cytoscape visualization of the co-expression network in the blue module. Figure S7. Alignment of the AtruNAC36 and AtNAC036 protein sequences. Five subdomains are labeled with red boxes. Asterisks represent the LVFY motif. Figure S8. Relative *AtruNAC36* expression levels in transgenic plants. Table S1. Primers used in this study. Table S2. Overview of the RNA-seq data for *A. truncatum* under drought conditions. Table S3. Identified DEGs in *A. truncatum* under drought conditions. Table S4. Transcription factor genes among the identified DEGs. Table S5. Selection of DEGs in *A. truncatum* under drought conditions for the GO enrichment analysis. Table S6. Selection of DEGs in *A. truncatum* under drought conditions for the KEGG enrichment analysis. Table S7. Genes in different clusters. Table S8. KEGG enrichment analysis of the DEGs in different clusters. Table S9. Genes in co-expression networks.
