# Supplementary figures and images for: Transcriptional Regulation of the Acer truncatum B. Response to Drought and the Contribution of AtruNAC36 to Drought Tolerance
Source: Antioxidants (Basel). 2023 Jun 24;12(7):1339. doi: 10.3390/antiox12071339 (PMC10376542; doi:10.3390/antiox12071339)

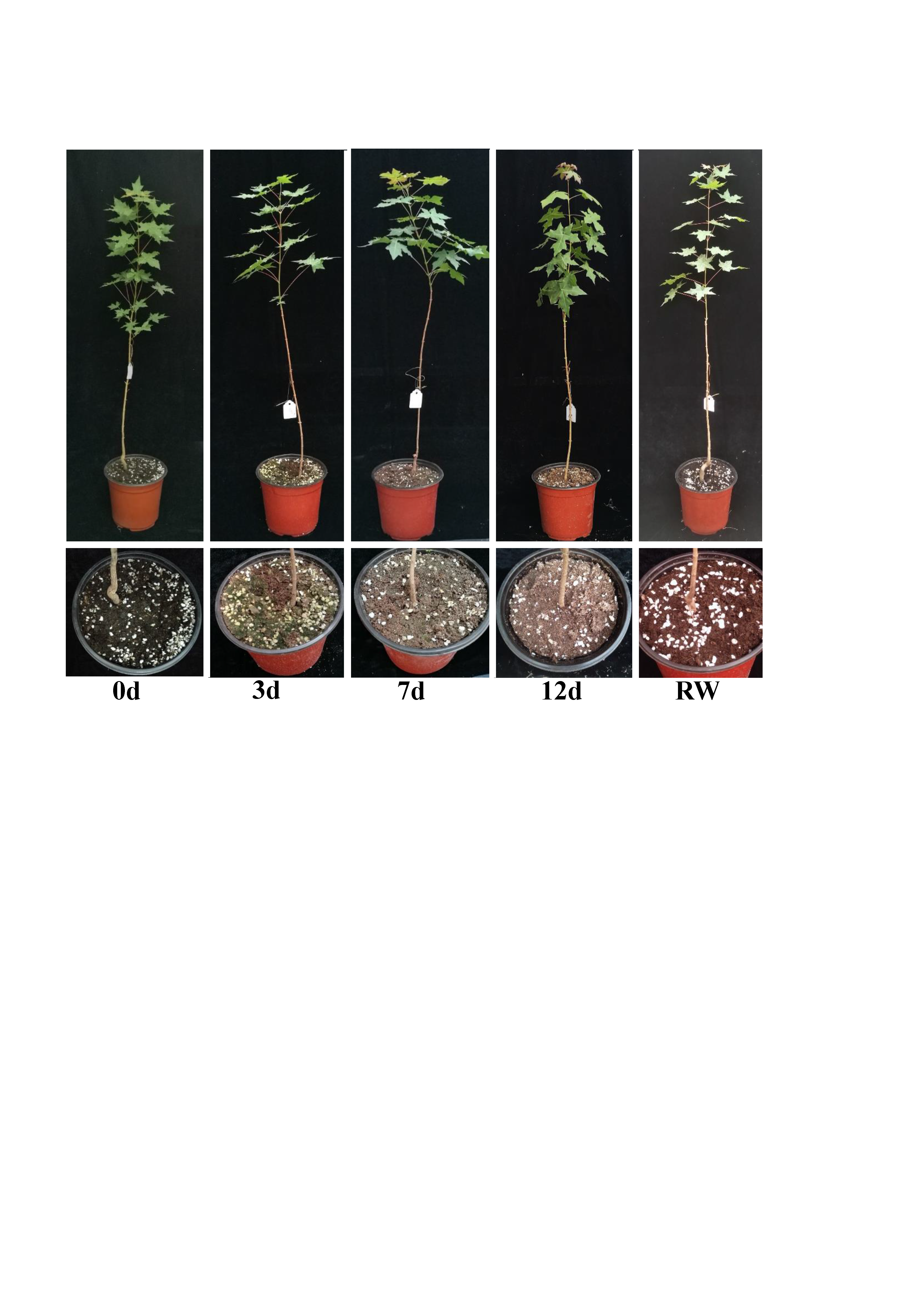

Supplement: Supplementary file 1 [file antioxidants-12-01339-s001.zip › Supplementary Files/Figure S1.tif]

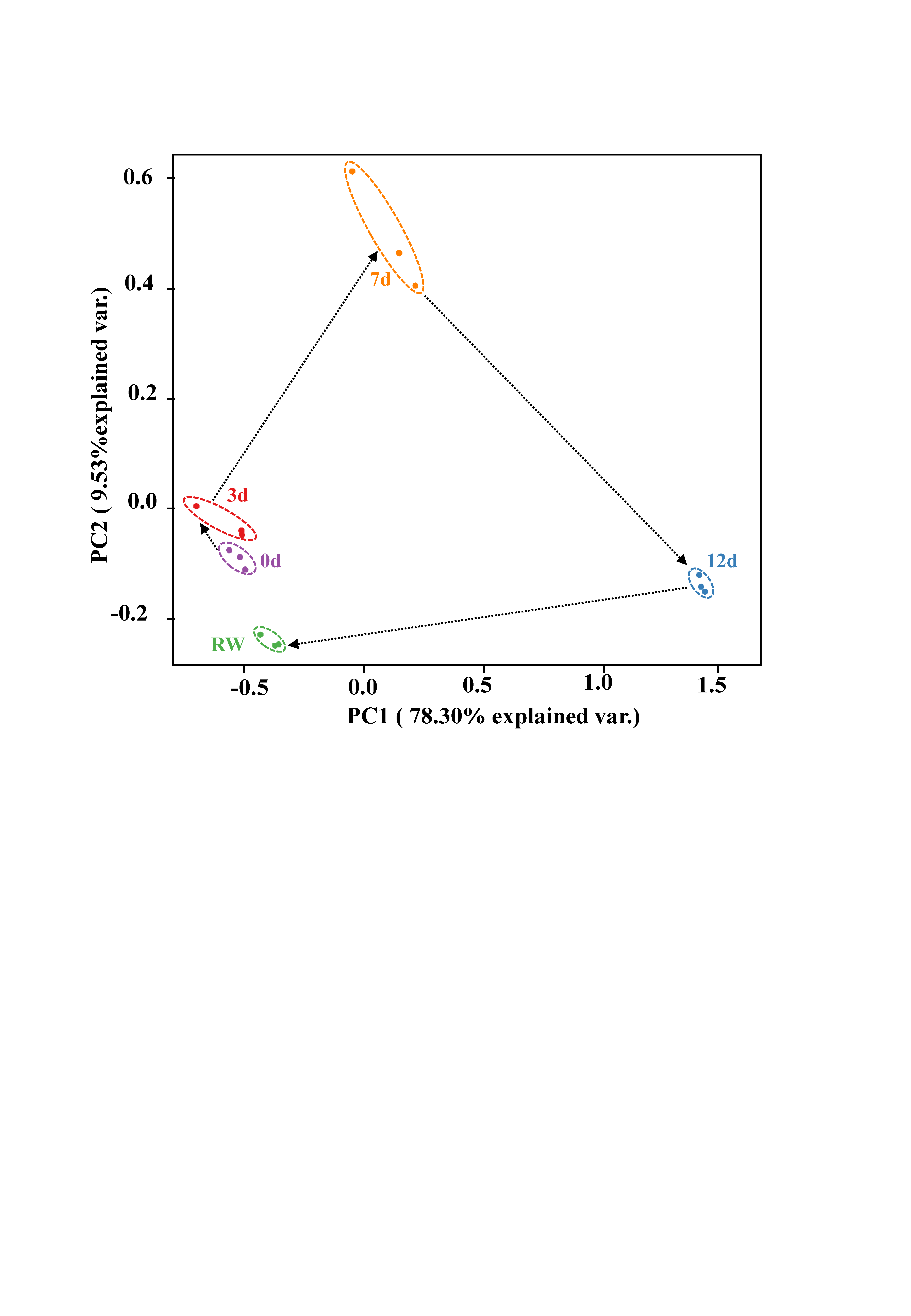

Supplement: Supplementary file 1 [file antioxidants-12-01339-s001.zip › Supplementary Files/Figure S2.tif]

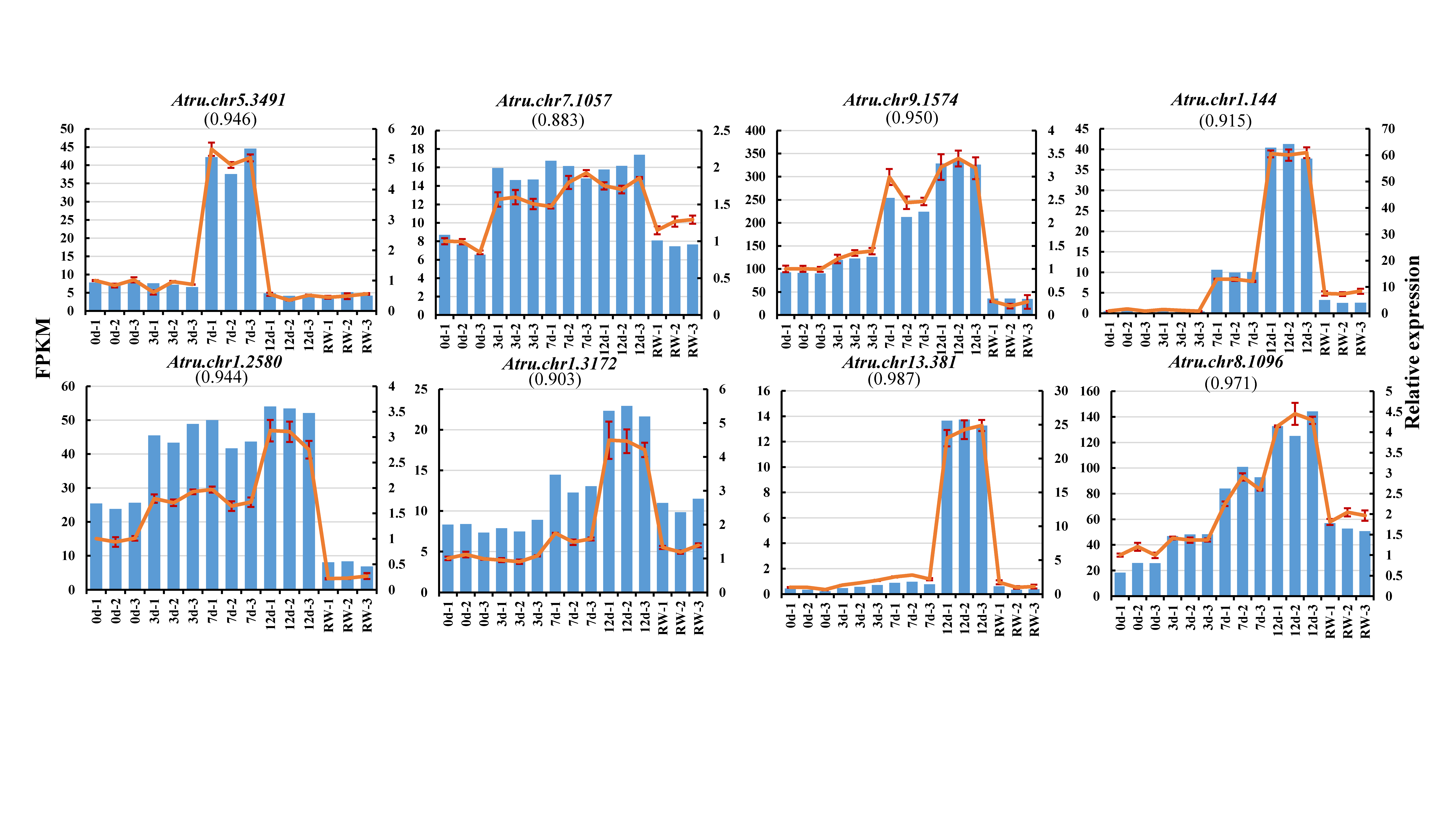

Supplement: Supplementary file 1 [file antioxidants-12-01339-s001.zip › Supplementary Files/Figure S3.tif]

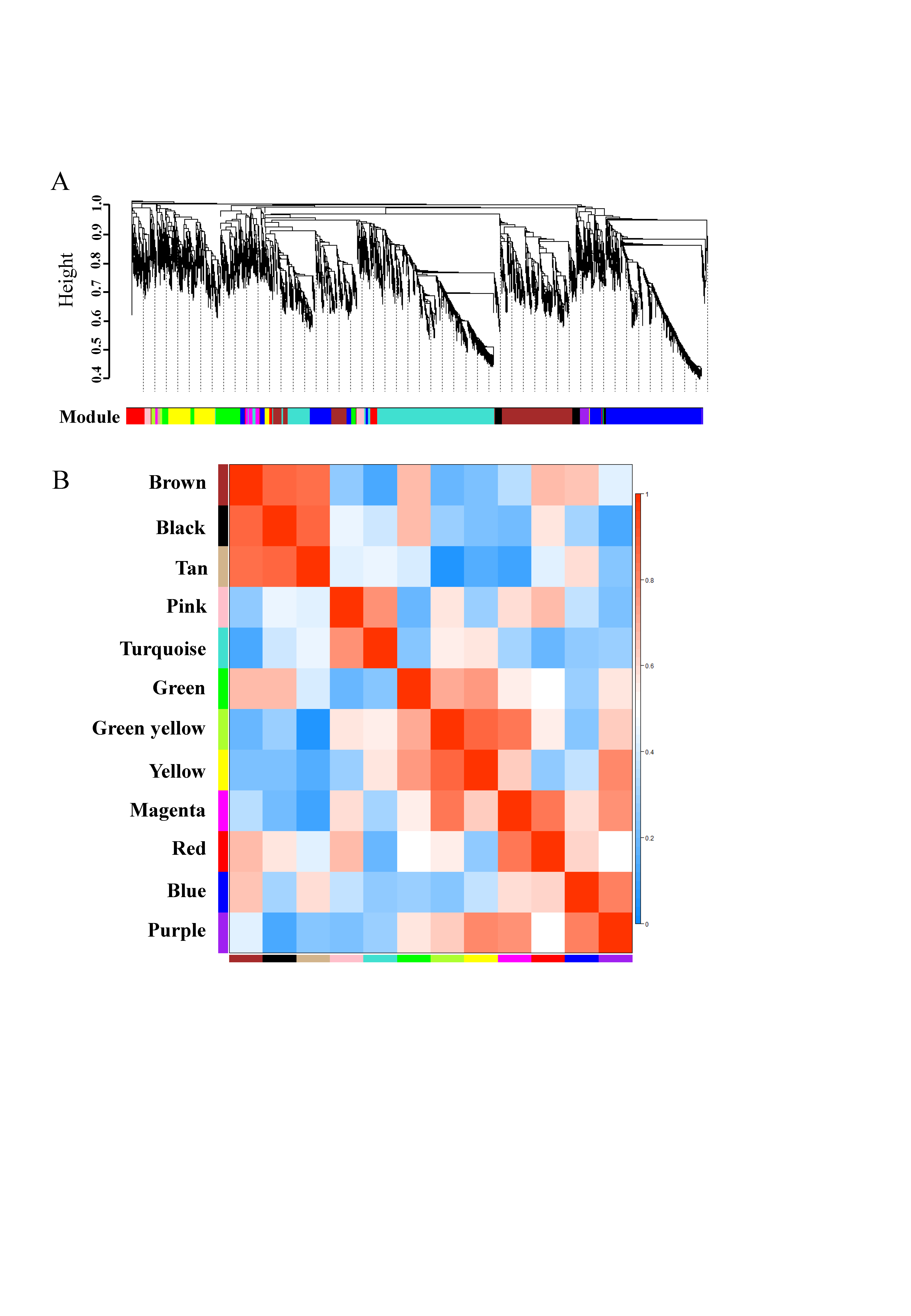

Supplement: Supplementary file 1 [file antioxidants-12-01339-s001.zip › Supplementary Files/Figure S4.tif]

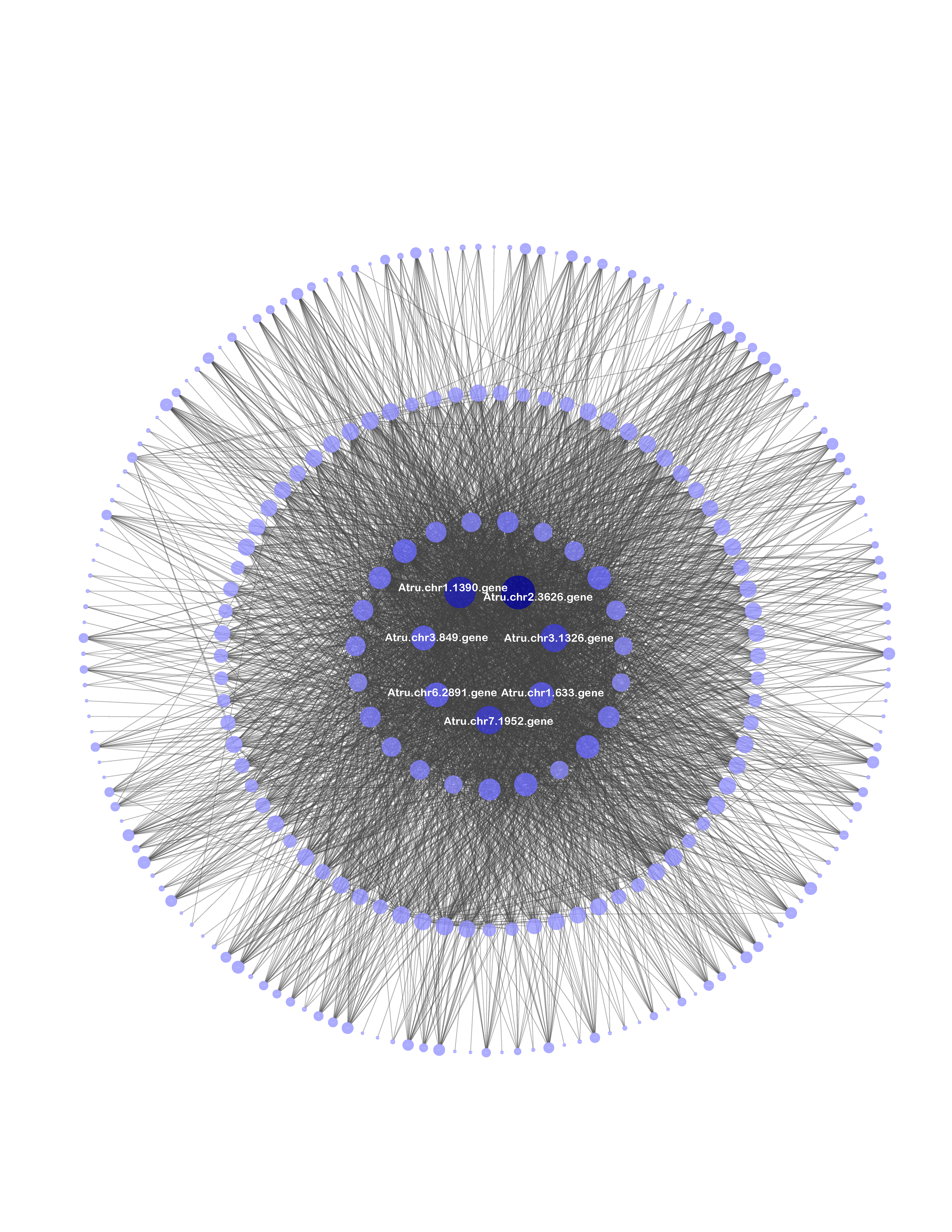

Supplement: Supplementary file 1 [file antioxidants-12-01339-s001.zip › Supplementary Files/Figure S5.tif]

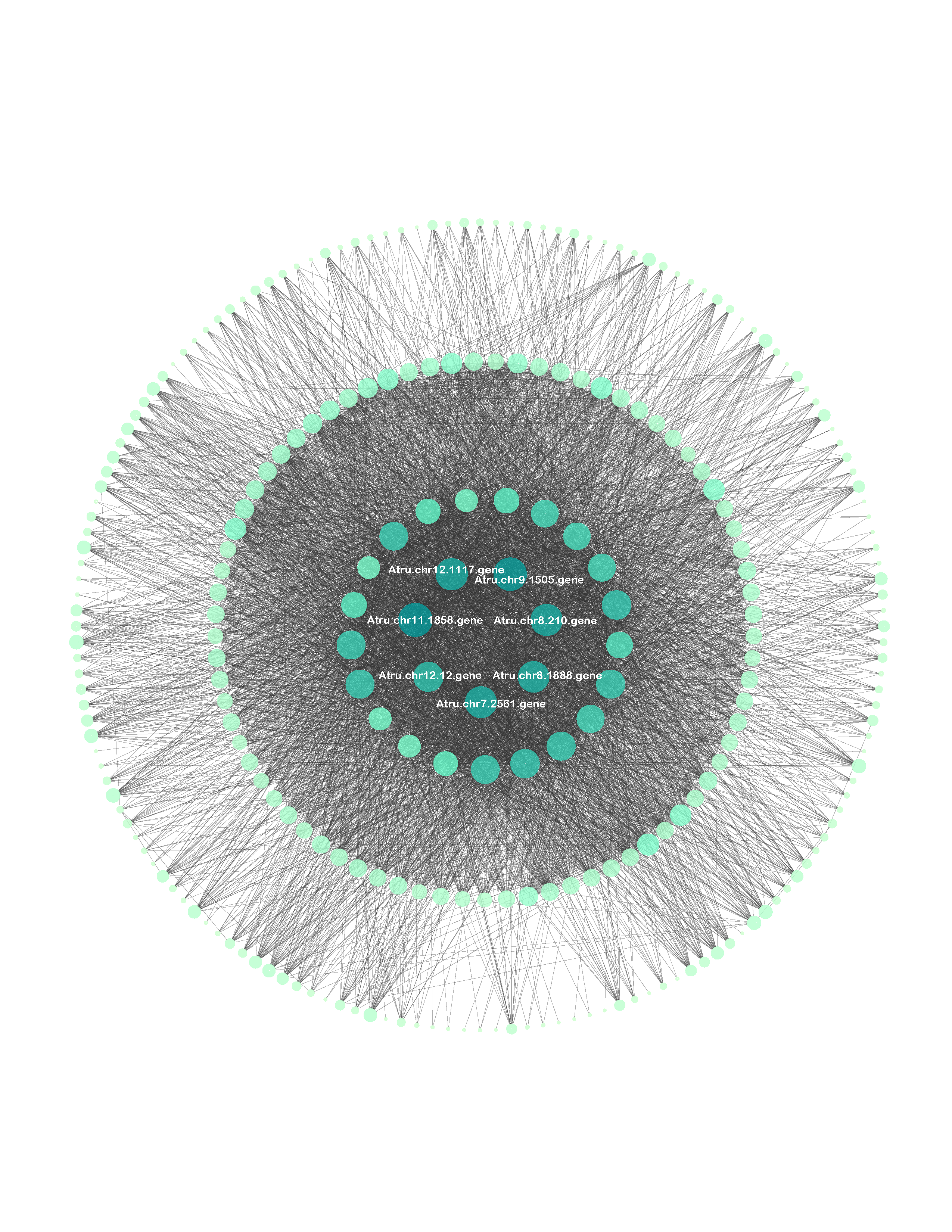

Supplement: Supplementary file 1 [file antioxidants-12-01339-s001.zip › Supplementary Files/Figure S6.tif]

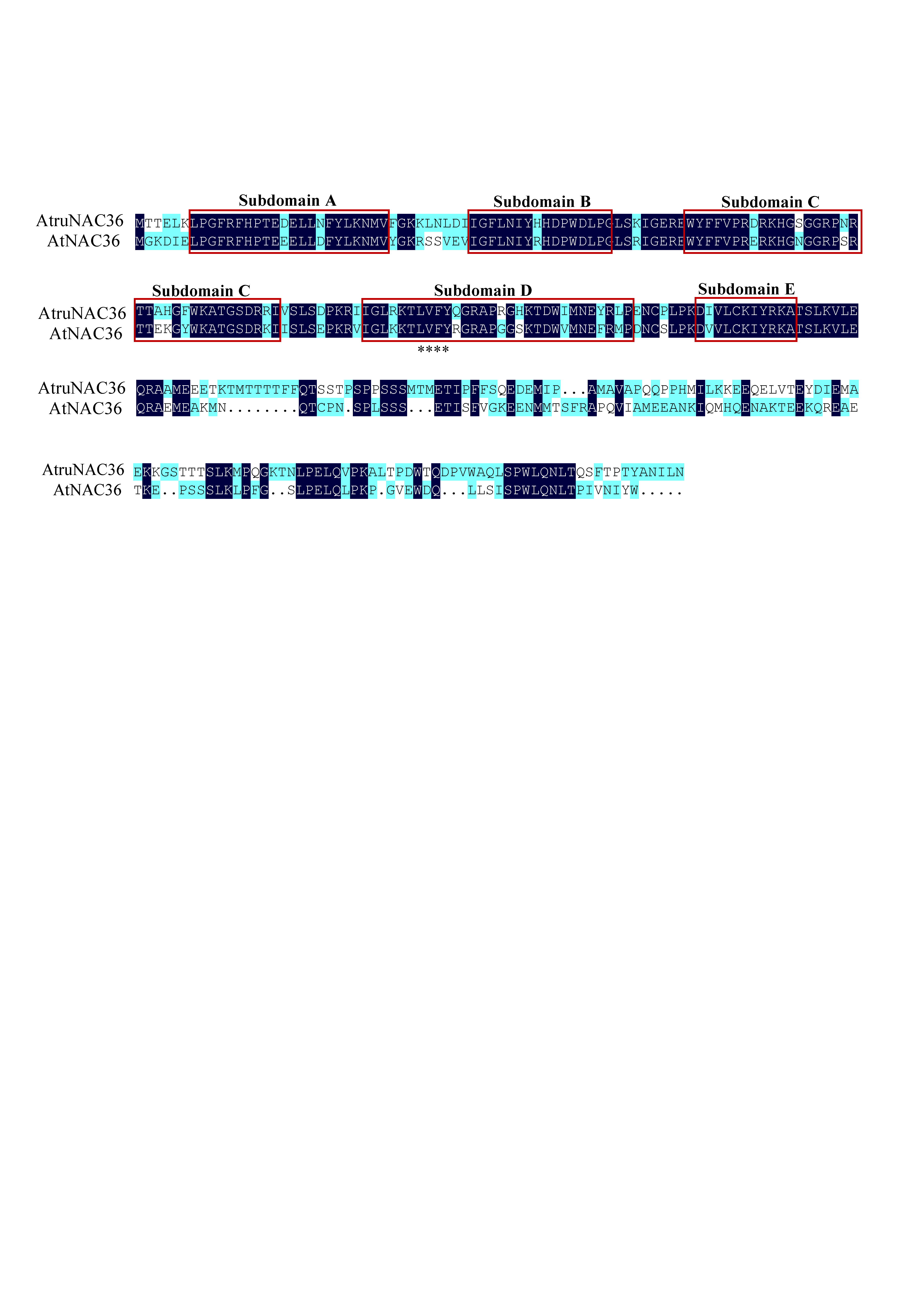

Supplement: Supplementary file 1 [file antioxidants-12-01339-s001.zip › Supplementary Files/Figure S7.tif]

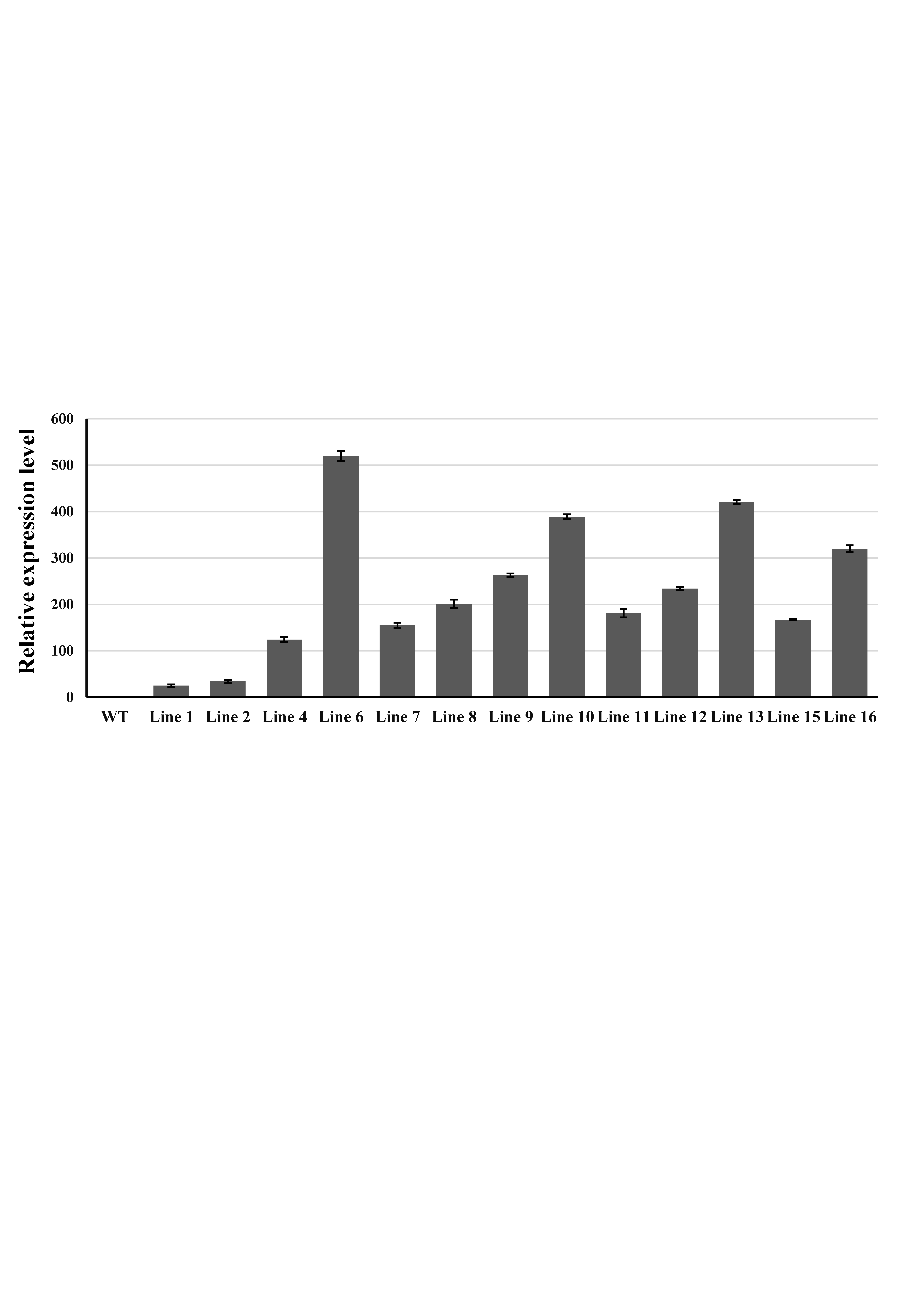

Supplement: Supplementary file 1 [file antioxidants-12-01339-s001.zip › Supplementary Files/Figure S8.tif]
